# Feasibility Study on S-Band Microwave Radiation and 3D-Thermal Infrared Imaging Sensor-Aided Recognition of Polymer Materials from End-of-Life Vehicles

**DOI:** 10.3390/s18051355

**Published:** 2018-04-27

**Authors:** Jiu Huang, Zhuangzhuang Zhu, Chuyuan Tian, Zhengfu Bian

**Affiliations:** 1 School of Environment Science and Spatial Informatics, China University of Mining and Technology, Xuzhou 221116, China; zzzhu@cumt.edu.cn (Z.Z.); 07143014@cumt.edu.cn (C.T.); zfbian@cumt.edu.cn (Z.B.); 2 State Key Laboratory for Geomechnics and Deep Underground Engineering, Xuzhou 221116, China

**Keywords:** 3D-thermal infrared imaging, microwave heating effect, polymer recognition

## Abstract

With the increase the worldwide consumption of vehicles, end-of-life vehicles (ELVs) have kept rapidly increasing in the last two decades. Metallic parts and materials of ELVs can be easily reused and recycled, but the automobile shredder residues (ASRs), of which elastomer and plastic materials make up the vast majority, are difficult to recycle. ASRs are classified as hazardous materials in the main industrial countries, and are required to be materially recycled up to 85–95% by mass until 2020. However, there is neither sufficient theoretical nor practical experience for sorting ASR polymers. In this research, we provide a novel method by using S-Band microwave irradiation together with 3D scanning as well as infrared thermal imaging sensors for the recognition and sorting of typical plastics and elastomers from the ASR mixture. In this study, an industrial magnetron array with 2.45 GHz irradiation was utilized as the microwave source. Seven kinds of ELV polymer (PVC, ABS, PP, EPDM, NBR, CR, and SBR) crushed scrap residues were tested. After specific power microwave irradiation for a certain time, the tested polymer materials were heated up to different extents corresponding to their respective sensitivities to microwave irradiation. Due to the variations in polymer chemical structure and additive agents, polymers have different sensitivities to microwave radiation, which leads to differences in temperature rises. The differences of temperature increase were obtained by a thermal infrared sensor, and the position and geometrical features of the tested scraps were acquired by a 3D imaging sensor. With this information, the scrap material could be recognized and then sorted. The results showed that this method was effective when the tested polymer materials were heated up to more than 30 °C. For full recognition of the tested polymer scraps, the minimum temperature variations of 5 °C and 10.5 °C for plastics and elastomers were needed, respectively. The sorting efficiency was independent of particle sizes but depended on the power and time of the microwave irradiation. Generally, more than 75% (mass) of the tested polymer materials could be successfully recognized and sorted under an irradiation power of 3 kW. Plastics were much more insensitive to microwave irradiation than elastomers. With this method, the tested mixture of the plastic group (PVC, ABS, PP) and the mixture of elastomer group (EPDM, NBR, CR, and SBR) could be fully separated with an efficiency of 100%.

## 1. Introduction

Since the beginning of the 21st century, polymer technologies have developed rapidly and been implemented in many fields of industry, which has significantly reformed the behaviors both in production and consumption [1,2,3]. Especially in the vehicle industry, huge amounts of polymer materials including plastics and elastomers have been utilized to take the place of metallic materials in vehicles in order to lighten the weight of cars and further reduce fuel consumption as well as the emissions of pollutants and greenhouse gases [4,5,6,7,8]. In the EU, vehicle land transportation contributes about 20% of the EU’s total CO_2_ emissions, and the CO_2_ emissions from land transport increased by about 23% from 1990 to 2010, which has been estimated to keep increasing in the future [5,7]. China has also had a rapid development both in vehicle production and consumption. In the last decade, the Chinese automotive industry had an annual average growth rate of about 20–25%. In 2014, the total vehicle production reached about 24 million, which made China the largest automobile producer and consumer in the world [9,10,11].

Transport is one of the major sources where greenhouse gas emissions are still rising. Currently, light-duty vehicles (LDV)—cars and vans—contribute around 15% of the EU’s CO_2_ emissions in land transport. Heavy-duty vehicles (HDV)—trucks and buses—contribute about 25% of the EU’s CO_2_ emissions in land transport and occupy about 6% of the EU’s CO_2_ emissions. Therefore, the EU commission has set corresponding legislation on emissions targets for both new passenger cars and van fleets [1,5,11,12]: For passenger cars, manufacturers are stipulated to produce new cars with emissions less than an average of 130 gCO_2_/km until 2015, and less than 95 gCO_2_/km until 2020. However, in 2007, the average emissions of passenger cars was about 160 gCO_2_/km and in 2011, this value was about 135.7 gCO_2_/km. According to fuel consumption, the target in 2015 was approximately 5.6 L/100 km of petrol and 4.9 L/100 km of diesel. The targets until 2020 are 4.1 L/100 km of petrol and 3.6 L/100 km of diesel.

For vans, the emissions reduction target was 175 gCO_2_/km until 2017 and 147 gCO_2_/km until 2020. In 2007, the average emission was about 203 gCO_2_/km and in 2010, this value was about 181.4 gCO_2_/km. Towards fuel consumption, the 2017 target was approximately 7.5 L/100 km of petrol and 6.6 L/100 km of diesel. The target until 2020 is 6.3 L/100 km of petrol and 5.5 L/100 km of diesel.

**Figure 1:**
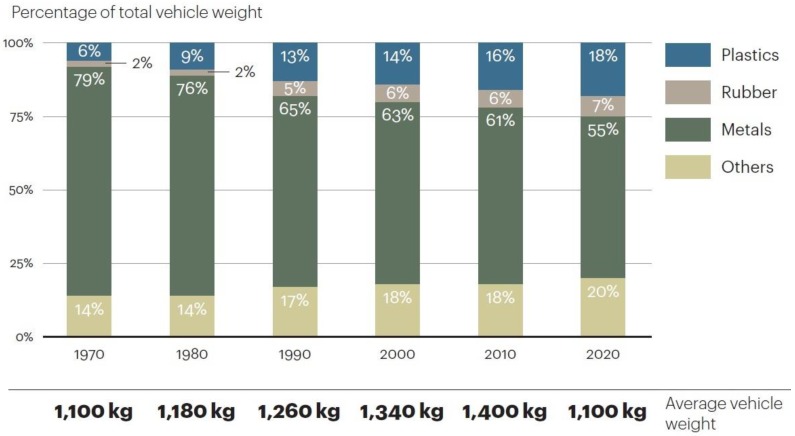
Material consumption of vehicles.

In order to fulfill the requirements of the CO_2_ emissions reduction, the weight reduction of the entire vehicle is an effective method. Therefore, more polymer materials have been developed and utilized in vehicles. With a weight reduction of 10%, fuel consumption can be decreased by about 6–8%, and every 100 kg of polymer materials can replace 200–300 kg of metallic materials. The application of polymers in passenger cars is higher than other kinds of vehicles, for example, the polymer application in a Santana (VW, Wolfsburg, Germany) is about 110 kg and in a VW Audi A6^©^ it is about 145 kg. Furthermore, in mini buses and trucks, it ranges between about 150 kg (Styer1491^©^, MAN, Munich, Germany) and 220 kg (Iveco ©, VOLVO, Gothenburg, Sweden). In some newly designed cars, e.g., the Audi A2^©^, the polymer parts represent a total of 300 kg, which is 28.8% of the total mass. This tendency can be shown by the vehicle material consumption in Figure 1 [3].

By 2020, the polymer material in vehicles is estimated to become more than 25% by mass. However, the increase in polymer utilization has caused serious problems in the disposal and recycling of end-of-life vehicles (ELVs) since the average life of vehicles is only about 8–15 years. To address this problem, nowadays most countries with vehicle industries have been encouraged to develop mechanisms and technologies for the reuse, recovery, and recycling of ELVs through legislation [1,2,3,4,10].

**Figure 2:**
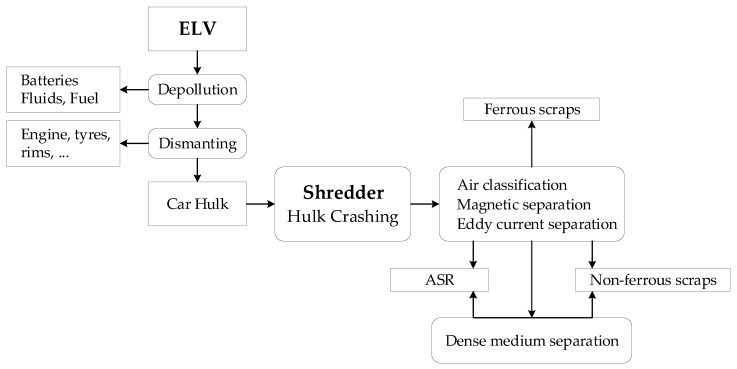
Recycling process of ELVs.

**Figure 3:**
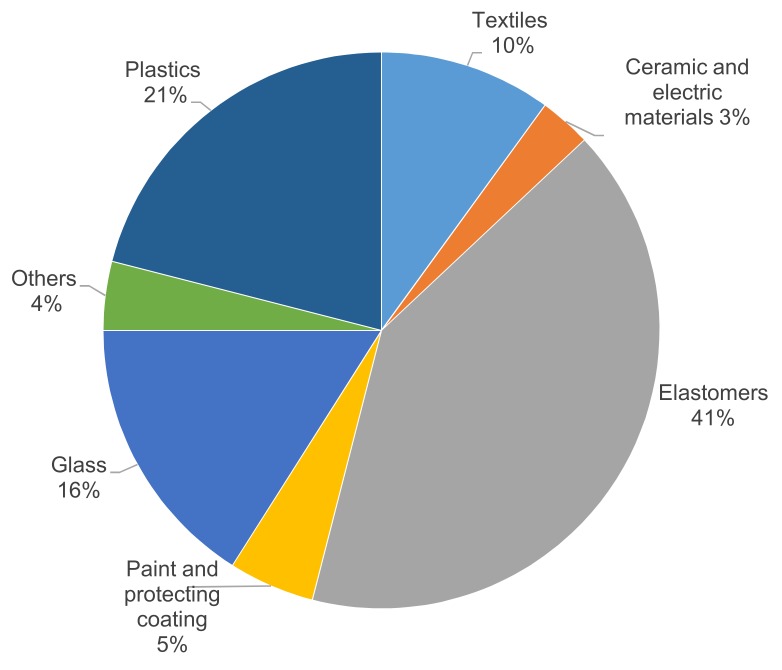
Ingredients of ASRs.

The actual recycling process of ELVs is as follows: first, ELVs arrive at an authorized treatment facility (ATF). There they are de-registered and de-polluted by the removal of batteries, tires, fluids, lubricants, and other hazardous and toxic materials. Then, they are further manually dismantled for reusable or recyclable parts [12,13,14,15,16,17,18,19]. After de-pollution and dismantling, the remaining vehicle-hulks are sent to a shredder where they are crushed into a mixture of small pieces. Then, the ferrous scraps are separated with a purity of more than 99% from the main stream by a magnetic sorter [1,4,10,16]. Next, non-ferrous fractions are sorted by an eddy current separator, which takes up to 6.7% of the original ELV mass [12,16]. After recovering the ferrous and nonferrous components, the rest of the waste stream still represents 15–25% of the original ELV mass and is known as “Automobile Shredder Residues” or “ASR”. Figure 2 summarizes the recycling process of ELVs:

With the increase of polymer utilization, the residual fraction of ASR is expected to increase in the future. Due to their high heterogeneity and hazardous ingredients, ASRs are nowadays commonly disposed of in landfill sites. ASRs contain more than 60% of the total weight of plastics and elastomers. Figure 3 shows the ingredients of ASRs as referred to in different studies [1,2,5,11,15,20].

ASRs contain many types of pollutants such as heavy metals and chlorine. Therefore, thermal recovery processes like pyrolysis, gasification, and incineration could bring new risks to the environment. The main automotive industry countries have created legislation for the material recycling of ELVs and ASRs. The EU Directives 2000/532/EC, 2001/18/EC2, 2001/119/EC3, and 2001/573/EC4 regulate that from the beginning of January 2015, ELVs should achieve a comprehensive recovery rate of 95% (weight), among which the recycling rate for secondary raw material must achieve 85% (mass) within the EU countries.

In China, the National Development and Reform Commission Proclamation 2006/9 also regulated that the recovery rate of ELVs needed to reach at least 95% by 2017, among which the recycling and reuse rate for secondary raw materials must represent at least 85%. In order to fulfill the requirements of these directives, the recycling of ASRs is the only effective method. Polymers occupy the greatest proportion of ASR ingredients and have stable properties against aging and corrosion, which are appropriate for producing secondary raw materials [1,4,5,20].

**Figure 4:**
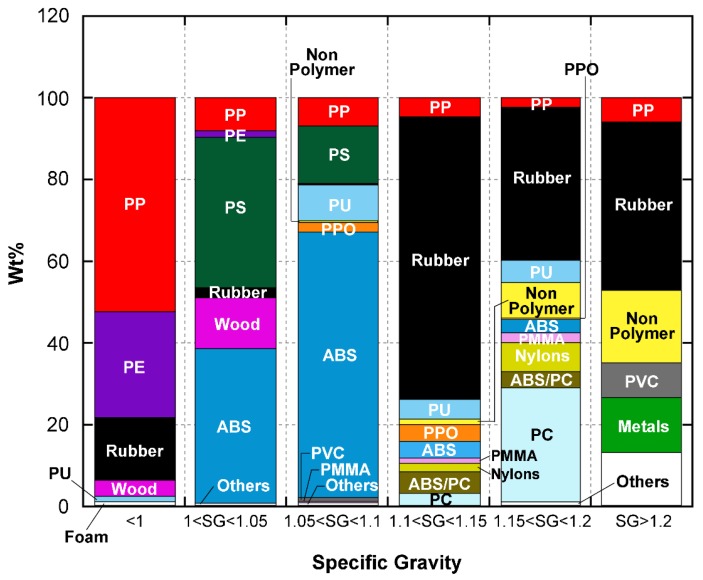
Flotation separation of ASR polymers.

Currently available post-shredding-technologies (PSTs) could realize the recycling of metallic, woody, and glass materials. The only technique which is effective for the separation of polymers is the sink-flotation method. However, this can only sort the ASR polymers into different mixtures with corresponding ranges of densities (specific gravities), as shown in Figure 4 [17,18,20,21].

Figure 4 shows that although some types of ASR polymers are significantly concentrated in a specific range of densities, like PP in the range of <1 g/m^3^, ABS in the range of 1.05–1.1 g/m^3^, and rubber in the range of 1.15–1.2 g/m^3^, the sorting products are still mixtures of several materials. In fact, the only successfully sorted pure material is PVC, which has a specific density of more than 2 g/m^3^.

The incomplete sorting of ASR polymers still limits their utilization as secondary raw materials. Although there have been some examples of successful implementation like the VW-SiCON process, R-Plus process, etc., none of these technologies has realized yet the material regeneration of ASR polymers. Hence, novel methods and technologies using corresponding force fields (gravity, magnetic, etc.) that are independent of traditional methods urgently need to be developed to produce secondary raw polymer materials with high purities [1,5,10,18,21].

Since the last decade, a new kind of material recognition and sorting mechanism by using modern sensor technologies, which are generally called sensor aided/based sorting (SAS/SBS) methods, has been rapidly developed. This mechanism is defined as single grain separation by using the contact-free detection of externally identifiable and measurable feature parameters. With the principles of SBS, several kinds of polymer recognition and sorting methods have been developed such as visual sorting, near infrared (NIR) and middle infrared (MIR) sorting, tribo-electrification sorting, etc. However, these principles are still unusable for ASR polymer mixtures. Visual sorters are only available for color detection, not for homochromatic features, and more than 95% of vehicle polymer materials are black; the NIR spectral analysis method is able to recognize most kinds of polymers with adequate precision, but it is unavailable for black and darkly dyed polymers, which absorb nearly 100% of NIR irradiation and result in inadequate reflection. The MIR spectral method can make sufficient reflected signals on the surface of black polymers, but it needs a very long irradiation time (several minutes), which strictly limits its industrial utilization. Furthermore, vehicle plastics are always modified and reinforced by padding with different additive agents like talc, carbon black, etc. These additives have a serious influence on the tribo-electrification properties of polymers [3,10,18,20,21,22].

In the last decade, sensor-based sorting by using microwave induced thermal imaging has been developed and implemented in sorting materials with microwave sensitivity. Microwaves are electromagnetic waves in a frequency range of 300 MHz to 300 GHz, which are always divided into several bands (S, X-band, etc.). S-band microwave radiation has been proven to be able to heat dielectric materials rapidly and homogeneously from the inside to outside as the sensitive parts to microwave irradiation are the polar molecules of materials. Several companies have developed vehicle elastomer and foam part production technology using microwave irradiation [23,24,25,26,27,28].

With the different molecular arrangements and structures of polymers, the heating effects are also different, which leads to temperature variations in irradiated materials. Particularly polar molecules, i.e., the additive agents used for the modification and reinforcement of polymers are very sensitive to microwaves, and can directly demonstrate the corresponding heating effect distributions, which could be utilized for the classification of specific kinds of materials [23,26,29,30]. With modern thermal imaging technology, the heating effect on polymers can be detected with high precision.

Microwaves heat materials that contain specific concentrations of polar molecules: when the dielectric of the polar molecules is placed in the microwave electromagnetic field, a dipole or an existing dipole rearrangement will be formed in the dielectric material. In the high-frequency alternative electromagnetic field, the movement orientations vary with a frequency of billions of times per second, which leads to strenuous movements and collisions between the polar molecules, which further cause a “stirring” effect at the molecular level [26,31,32]. This effect results in the generation of large amounts of heat which can achieve the direct conversion of electrical energy to inner heat. The thermal energy in the medium increases the temperature of the materials, and it can be seen that the microwave heat comes from the loss of electric field energy inside dielectric material [33]. Vehicle polymers consist of different kinds of elastomers and plastics, which are significantly different in polarity. This difference of polarities can be used for the sorting and separation criteria of ASR polymers. The difference of material polarities can be deduced by variations in the microwave heating effect, which can be easily acquired through modern infrared imaging technology [34,35].

In this research, we studied a novel method using S-band microwave irradiation together with 3D imaging as well as thermal imaging sensors to recognize and sort the plastic and elastomer materials in ASR mixtures. In this study, an industrial microwave emitter array with 2.45 GHz/9 kW was utilized as the irradiation source. Seven kinds of vehicle polymer materials were tested: three kinds of plastics: polypropylene (PP), polyvinyl chloride (PVC), and acrylonitrile butadiene styrene (ABS); and four kinds of elastomers: styrene butadiene rubber (SBR), chloroprene rubber (CR), nitrile butadiene rubber (NBR), and ethylene-propylene-diene monomer (EPDM).

## 2. Materials and Methods

### 2.1. Principle of Microwave Heating

Microwave irradiation can be reflected, absorbed, or penetrate the surfaces of materials and has nearly no energy loss in transmitting insulators. Conductors such as metals can reflect microwaves and result in no transmission. Water, metal oxides, and carbon-based materials can cause high energy loss during transmission, and these kinds of materials are called dielectric materials [23,24,25,26].

The mechanism of microwave field energy loss caused by transmission in dielectric materials is as follows: in an alternative electromagnetic field, polarization appears in dielectric molecules where the dipole and interfacial polarization time is about 10^−9^–10^−12^ s. This period coincides with the frequency domain of microwaves, which cause the molecule polarization movements which lag behind the frequency change of the alternating electromagnetic field, and further lead to the dielectric loss of microwave energy. The dielectric properties of materials determine their energy absorption, i.e., the heating effects under microwave irradiations, which can be described by using complex permittivity *ε* as [31,33]:

(1) $$\varepsilon =\;\varepsilon_{0}\varepsilon_{r}=\;\varepsilon_{0}\;\left(\varepsilon_{r}^{\prime }-\;i\varepsilon_{r}^{\prime \prime }\right)$$

(2) $$\tan\;\delta \;=\;\frac{\varepsilon_{r}^{\prime \prime }}{\varepsilon_{r}^{\prime }}$$

where *ε*_0_ is the vacuum permittivity with a constant value of 8.854 × 10^−12^ F/m. *ε_r_* is the complex permittivity where $\varepsilon_{r}^{\prime }$ is its real part, and represents the dielectric energy storage capacity through material molecule polarization. $\varepsilon_{r}^{\prime \prime }$ is the imaginary part, which represents the loss of electromagnetic energy inside the materials. tan *δ* is the tangent of the energy loss angle, which indicates the ratio of microwave energy loss to energy stored in the molecules’ polarization. Most of the commonly used materials have a very small value of vacuum permittivity, normally we use *ε_r_* and tan *δ* to describe the materials’ dielectric properties.

The electromagnetic energy loss due to the dielectric properties is absorbed by the materials and finally results in an increase in temperature. Polymers including plastics and elastomers are mostly insensitive to microwave irradiation, but the polymers used to produce vehicle parts are always modified or reinforced for better mechanical properties. The additive agents used for modification are carbon-based or mineral materials, which are specifically formulated for the corresponding polymer and are highly sensitive to microwaves. Therefore, we can distinguish different vehicle polymers by detecting their heating effects under microwave irradiations since different kinds of polymers have different modification and reinforcement formulations. The direct measurement of tan *δ* is hard since it is very difficult to measure the energy loss of microwaves before and after transmission in materials. However, the effect of tan *δ*, i.e., the temperature increase, can be easily measured by using modern sensor technologies such as infrared thermal imaging cameras.

Microwave heating efficiency can be described by using the average power absorption density *P_d_* as [33]:

(3) $$P_{d}\left(z\right)=\frac{\omega }{c}\times \frac{4\varepsilon_{r}^{\prime \prime }S_{0}e^{-2\alpha z}}{\sqrt{\varepsilon_{r}^{\prime }{}^{2}+\varepsilon_{r}^{\prime \prime }{}^{2}}+\sqrt{2\left(\sqrt{\varepsilon_{r}^{\prime }{}^{2}+\varepsilon_{r}^{\prime \prime }{}^{2}}+\varepsilon_{r}^{\prime }\right)+1}}$$

where *S*_0_ is the average power density of microwave irradiation; ***c*** is the vacuum light velocity; *ω* is the angular frequency of microwave; *α* is the microwave attenuation coefficient; and *z* is the transmission depth of microwave. According to Equation (3), the transmission thickness of the tested object can have an influence on heating homogeneity. However, as related studies have pointed out, for normal dielectric polymer materials, with a transmission thickness less than 10 mm and an irradiation power larger than 9 kW as well as an irradiation time of less than 30 s, the heterogeneity of temperature increasing from the inside to the surface is always less than 0.1 °C, which can be ignored for the purposes of this study. All of the tested polymer scraps came from crushed vehicle parts; due to the purpose of vehicle lightening, most vehicle parts had a hollow structure with a small thickness. Hence after crushing, the scraps had a flaky structure with thicknesses around 3–8 mm. Polymer recognition by detecting the heating effect of microwaves on flaky scraps was appropriate for our research.

### 2.2. Experimental Facility Installation

In our research, an industrial conveyor microwave facility with a frequency of 2.45 GHz (wavelengths of approximately 12 cm) was used. This frequency corresponds to the most utilized heating frequency in the S-band. The conveyor microwave facility was set up by the company Media^©^ in December 2016 for our research. This facility has a 2 m long chamber which is completely surrounded by stainless steel to confine all the irradiation inside the chamber. Its interior walls are porous; below the conveyor belt, ceramic plates were installed to absorb the reflected microwave radiation from the stainless-steel wall.

Above the inner conveyor, there are six magnetrons with a frequency of 2.45 GHz installed on the top of the microwave chamber in one row. In this research, we used three magnetrons. Each magnetron had a maximum output of 3 kW that could be varied in 1 w increments. The maximum capacity of the microwave radiation was 9 kW, and the homogeneity of microwave irradiation was insured by the facility supplier.

**Figure 5:**
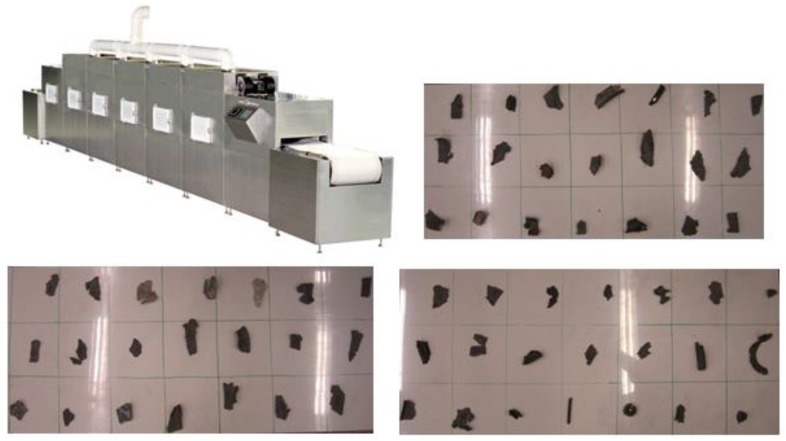
Installation of conveyor microwave facility and the tests of polymer scraps.

The conveyor belt was made of a beige textile with a width of 50 cm. This textile ensured that the conveyor belt would not be heated within an irradiation period of several hours. The velocity of the conveyor belt could be adjusted from 0.2 m/s to 2 m/s, with an increment of 0.1 m/s to be varied. The whole installation of the facility and the tests of the polymer scraps are shown in Figure 5.

In this research, the velocity of the conveyor belt was set at 0.2 m/s, therefore the tested scraps were irradiated in microwave field for 10 s.

### 2.3. Sensor System Installation

**Figure 6:**
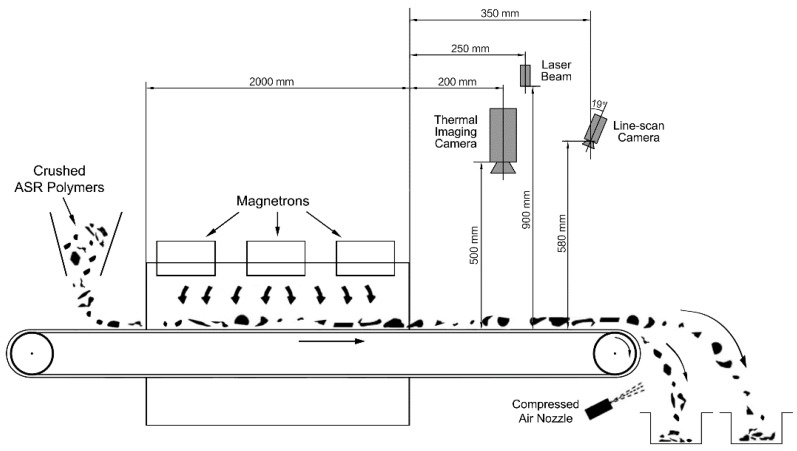
Installation of the sensor system on the conveyor microwave facility.

The sensor system was composed of a thermal imaging infrared camera and a 3D line scan camera together with a laser-triangulation setting. The design and installation of the sensor system is shown in Figure 6.

All the crushed ASR polymer scraps were designed to be dropped onto the conveyor individually by the vibration feeder, then they were carried to pass through the microwave irradiated area where they were heated. Above the exit of the microwave chamber, two sensors were installed to acquire the features of the tested scraps, then at the end of the conveyor, the recognized object scraps were sorted by compressed air separation from the main scrap stream.

The thermal infrared camera captured the temperature information of the single polymer scraps and saved them in a grayscale graph. By using this method, it was possible to acquire any pixel of temperature in the thermal images [34,35]. The infrared camera was a line scan camera with the model of “pyroline 128 ls/512 Hz” from the company DIAS Co. Ltd. (Dublin, Ireland), which is available for the temperature range of 0 to 80 °C with a resolution of 0.1 °C. The measurement uncertainly of this infrared camera was *σ* = 0.01 × T °C (T is the ambient temperature). The infrared camera had its CCD sensor in 128 pixels. Its properties were enough for the acquisition of the heating effect. The other information for positioning and image mask generation of the tested scraps were realized with the help of 3D scan sensor.

The 3D line scan camera was the optical sensor used to acquire the position and geometrical features of the tested scraps and provide masks that could be used to cover the thermal image for locating the tested scraps. The scrap positions acquired by this sensor were used to control the air nozzle to blast the corresponding scraps out of the main material stream.

**Figure 7:**
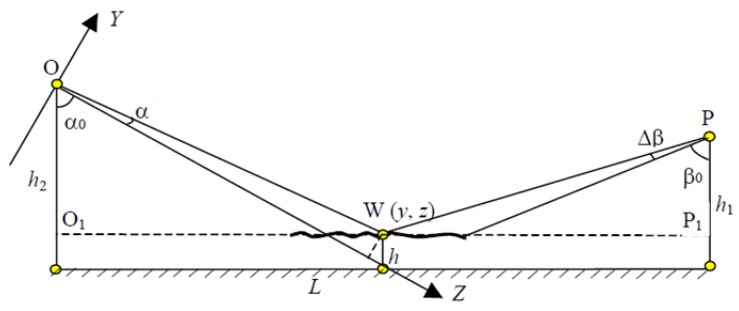
Mechanism of laser triangulation scanning.

The acquisition of the height information of the tested scraps was realized by the mechanism of 3D laser triangulation. The mechanism of the 3D camera triangulation measurement is shown in Figure 7. In Figure 7, *O* and *P* represent the focal point of the 3D scan camera and the position of the laser beam, respectively. In this research, we used only one laser beam to be emitted vertically to the conveying direction. *h*_1_ and *h*_2_ represent the heights of the 3D scan camera and laser beam, respectively. The height of a scrap’s surface point *W* could be set with *h*. The horizontal distance of the conveyor belt between the 3D scan camera and laser beam is *L*, where $L=\bar{O_{1}P_{1}}$. The installation angle between the lens axis and vertical direction is *α*_0_, and the angle between the line $\bar{OW}$ and the lens axis is *α*. *β*_0_ is the installation angle between the laser path and vertical direction, and Δ*β* is the laser path angle between the adjacent laser beams, here we had only one laser beam.

**Figure 8:**
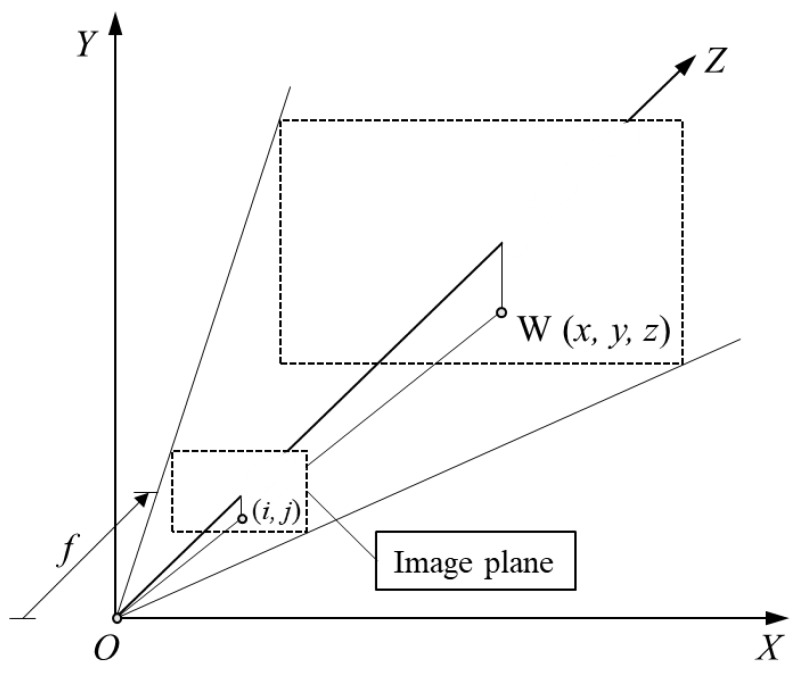
CCD imaging mechanism.

The installation of the scan camera, the laser beam, and the tested objects formed triangle structures, which constitute the 3D-laser-triangulation measurement. The laser beams were photographed with different grayscales by the line scan camera according to the height of the tested scrap surface, i.e., the distance between the camera lens and scrap surface. The distance *L* is known, the setting angles *α*_0_ and *β*_0_ are also given, and the angle *α* could be determined by focusing on the strike point of the laser beam. These geometrical parameters determined the size of a scrap triangle and gave its position. The imaging of the laser triangulation on the camera sensor is shown in Figure 8.

If *f* is the focal length of the camera and (*i*, *j*) is the pixel in the image plane that corresponds to a point *W*(*x*,*y*,*z*) on the specimen surface, then two similar triangles give the following equation:

(4) $$\frac{y}{j}=\frac{z}{f}$$

According to the laser triangulation mechanism and the triangles Δ*OO*_1_*W* and Δ*PP*_1_*W*, the following equation can be obtained:

(5) $$h=\frac{[L-h_{1}\tan(\beta +\Delta \beta )](f-j\tan\alpha_{0})-(j-f\tan\alpha_{0})h_{2}}{(f-j\tan\alpha_{0})\tan(\beta +\Delta \beta )+f\tan\alpha_{0}+j}$$

where the focal length of the 3D line scan camera is *f* and (*i*, *j*) is the pixel in the imaging area that corresponds to the point *W*(*x*,*y*,*z*) on the tested scrap. In Equation (2), only *j* is a variable that needs to be determined by the camera sensor. All the other parameters are given through the system structure. In this research, only one laser beam was utilized and set to irradiate vertically on the conveyor belt, hence the angles *β*_0_ and Δ*β* were zero. Then, Equation (2) can be rewritten as:

(6) $$h=\frac{L\;(f-j\tan\alpha_{0})-(j-f\tan\alpha_{0})\;h_{2}}{f\tan\alpha_{0}+j}$$

Equation (3) describes the measurement of the height information of the tested scraps, which could be combined with the shape and position features that are also acquired by the 3D line scan camera to generate the mask image. Masks were used to cover the thermal image in order to determine the useful area where the heated polymer scraps were presented.

The 3D line scan camera was an AT C3-1280-CL (BASLER, Ahrensburg, Germany) 16-bit line scan camera with a highest scan frequency of 47,000 lines/s and a resolution of 1280 pixels. The laser beam used for the 3D measurements was monochromic, with a wavelength of 660 nm.

### 2.4. Implemented ASR Polymer Materials in This Research

The ASR polymer materials came from diverse parts such as bumpers, side bumper slides, dashboards, fuel tanks, pipes, sealing elements, fenders, pedals, inner decorations, exterior parts, etc. In this research, the tested ASR polymers were sampled from a vehicle recycling facility located near the city of Nanjing. Due to the number of polymers utilized in vehicles, we sampled seven of the most commonly found materials directly from ELVs, including four kinds of elastomers: styrene butadiene rubber (SBR), chloroprene rubber (CR), nitrile butadiene rubber (NBR), and ethylene-propylene-diene monomer (EPDM); and three kinds of plastics: polypropylene (PP), polyvinyl chloride (PVC), and acrylonitrile butadiene styrene (ABS). All of the parts that belonged to those materials in single ELVs were sampled. The usage of tire rubber is normally much more than these kinds of polymers, but the tires are usually disassembled before being crushed as part of another recycling process. Tire rubber is rarely found in crushed ASRs. The absolute amount of PVC is normally minor in ASR mixtures, but due to its high toxicity to the environment, PVC must be removed as much as possible before landfilling. Therefore, PVC was also studied in this research.

Some of the vehicles were used for much longer than their legal lifetime. Hence, we sampled the seven kinds of materials from ELVs that were manufactured during two periods: 2000–2005 and 2006–2010, respectively. The average usage time of the ELVs was 12.7 years. The sample information is shown in Table 1.

Generally, there was no significant difference in either the absolute amounts or the proportional usages. Imported vehicles always utilize more engineering plastics than domestic vehicles. The seven kinds of typical materials accounted for more than 85% of the total polymer materials in ELVs. After crushing with a hammer mill, the particle size distribution of these scraps was 4–22 mm, and their thicknesses covered a range of 2.5–8.7 mm. The total mass of sample material utilized for this research was PVC 5.12 kg, ABS 4.78 kg, PP 4.36 kg, EPDM 5.77 kg, NBR 6.45 kg, CR 6.32 kg, and SBR 6.68 kg, respectively. All the tested samples had a water content of less than 1%.

## 3. Results and Discussion

In the experiments, according to the research purposes there were two approaches. In the first approach, the heating temperature of the different material scraps was determined. In the second approach, the corresponding material scraps were sorted by using the variations of their heating temperature features. All of the results are introduced in the following subsections.

### 3.1. Determination of Heating Effects

**Figure 9:**
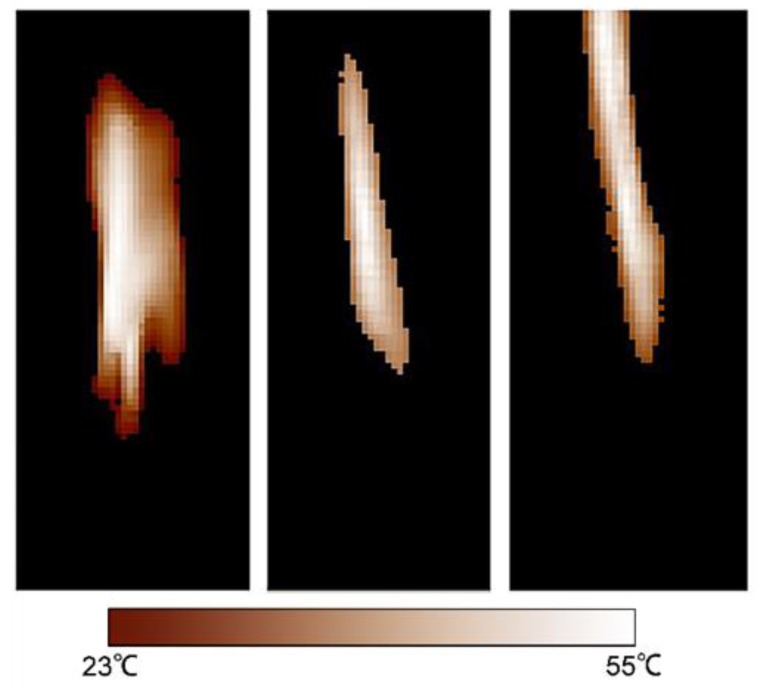
Pseudo-coloring of temperature infrared images.

The ambient temperature when the experiments were implemented was 18.5–27.5 °C. Before each experiment, the ambient temperature was measured as the basic temperature of the tested scraps and the lower threshold of the infrared imaging camera. All of the polymer scraps were tested to pass through the microwave irradiation field three times with the irradiation power of 3 kW, 6 kW, and 9 kW, respectively. The velocity of the conveyor belt was 0.2 m/s for all tests, i.e., the irradiation time was 10 s for all tested scraps. The temperatures of the heated scraps were acquired online by the infrared imaging camera. Figure 9 shows a pseudo-colored image of the microwave heating effect on a pixel scale on very small tested particles.

Figure 9 demonstrates the heating effect distribution on tested polymer scraps from pixel to pixel in pseudo-coloring, where the original image was illustrated in grayscale. A lighter color indicates a higher temperature. The pseudo color images obviously showed that the differences of temperature increase in different polymer materials. It was found that on the edges of the scraps, the temperatures were lower than in the middle of the scraps. The reason was that because the size of these scraps was too small (less than 5 mm), when they came out of the irradiation chamber, they were rapidly cooled down. Additionally, the infrared camera had to be placed at least 200 mm away from the exit of the microwave chamber to avoid the interference from microwave radiation on the infrared sensor. Therefore, the tested scraps needed to have relatively larger particle sizes. In this research, by using appropriate crushing velocities, we kept 95% (by mass) of the tested polymer scraps with particle sizes larger than 30 mm.

**Figure 10:**
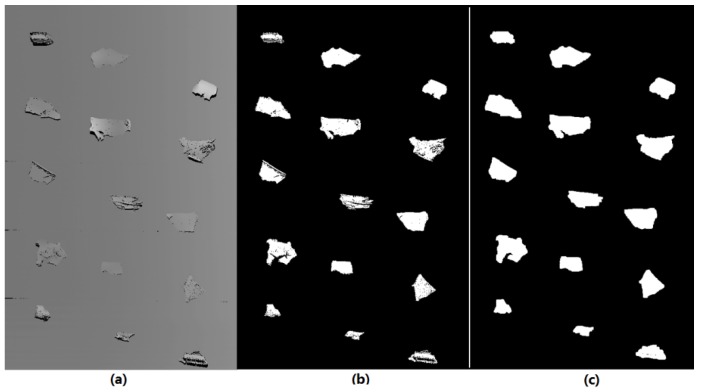
Process for producing of mask for infrared thermal images: (**a**) 3D scan grayscale image; (**b**) binary image of 3D scan image; and (**c**) prepared mask for infrared thermal image.

The infrared thermal imaging sensor could only record the temperature of its whole viewing area. In order to determine the interesting areas where the tested scraps lay on the conveyor, we used the 3D line scan camera to produce masks for the thermal images where the corresponding area of the tested scraps were illustrated using a binary mask image. This process is shown in Figure 10.

In the raw 3D line scan image, the height of the surface on which the laser beam actually stroked was illustrated with different grayscales. This grayscale image was 16-bit, which means that in each pixel, there were 2^16^ different grayscale values to describe the height of the scraps. Here, we selected the surface of the conveyor belt as the lower threshold plane of the grayscales, and then set all the grayscales, which were larger than the threshold, as equal to 1 (white), meaning the area with heights larger than the surface of conveyor belt i.e., the area of scraps. Grayscale values equal to/smaller than this threshold were set at 0 (black), which meant the area without the tested scraps. Therefore, we obtained a binary image of the 3D scan image, i.e., the position and geometrical area features of the tested scraps. This binary image was the mask image for the infrared thermal image.

As all of the tested scraps were black or dark dyed, therefore, some of their surfaces could not generate an adequate reflection of the laser beam and further caused black spots inside the classified object area on the binary image. Hence, we added more processing by using erosion algorithms to eliminate these spots inside the scrap areas, as illustrated in Figure 10c.

**Figure 11:**
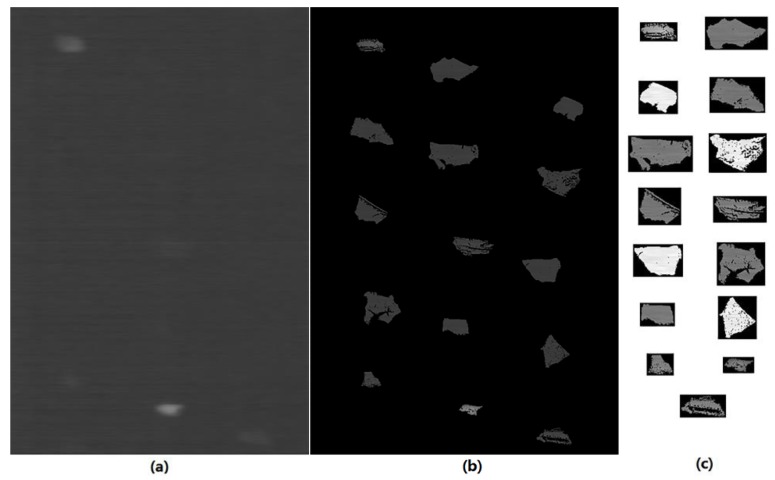
Temperature information acquisition from infrared thermal image: (**a**) original thermal image; (**b**) mask on the thermal image; and (**c**) segmented area of object scraps of thermal image.

By using the mask image, the corresponding areas on the thermal image that described the temperature information of the tested scraps could be segmented for further analysis. This process is shown by one test on an example material in Figure 11.

Figure 11 shows that, from the raw infrared thermal image, it was very difficult to determine the area of the tested objects, especially when their temperature increases were not significant and could not be distinguished by the human eye. With the help of the binary mask image, as shown in Figure 11b, the interesting areas where the tested scraps were located were easily determined and then the object areas were segmented from the thermal image for temperature feature extraction. In fact, the interesting areas were segmented according to their edges on the mask, here we had to show them clearly; hence we demonstrate these with a rectangular window in Figure 11c.

The temperature distribution features on the entire surface of the tested polymer scraps acquired by the infrared imaging camera were stored with a 5% percentile step, then they were analyzed and illustrated with Box-Whisker diagrams. In this diagram, the distribution of the acquired temperature data was illustrated with the minimal and maximum values and the 25%, 50%, and 75% percentiles. Of course, the percentiles could also be set arbitrarily from 1–100% according to the different data mining requirements.

### 3.2. Microwave Heating Effect on Tested Materials

**Figure 12:**
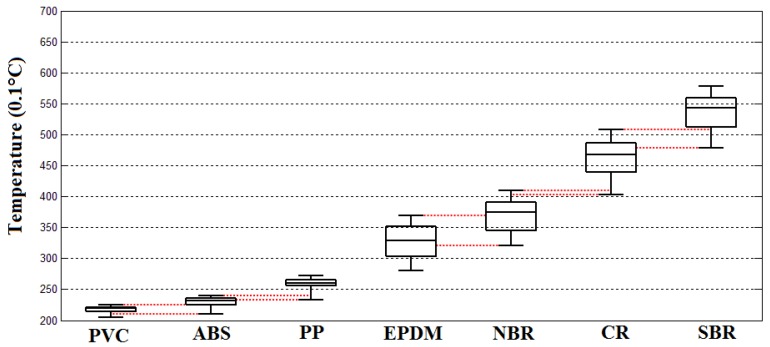
Distribution of temperature rises of the tested polymer samples under a microwave irradiation power of 3 kW.

**Figure 13:**
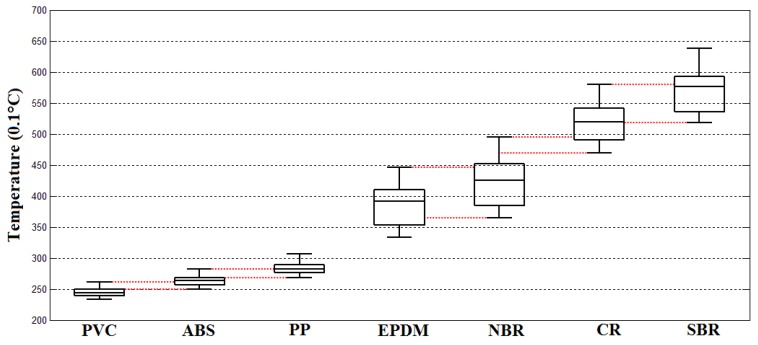
Distribution of temperature rises on tested polymer samples under microwave irradiation power of 6 kW.

The temperature rising features of the tested ELV polymer scraps including PVC, ABS, PP, EPDM, NBR, CR, and SBR under the irradiation power of 3 kW, respectively, are shown in Figure 12 as follows:

The ambient temperature when this experiment was implemented was about 19.5 °C with a 3 kW microwave irradiation of 10 s. The temperature distribution features of tested scraps were:

- The temperature distribution of the PVC scraps was 20.5–22.5 °C, the 25% percentile was about 21.5 °C, the 75% percentile was about 22 °C, and the median temperature was about 21.8 °C.
- The temperature distribution of the ABS scraps was 22–24 °C, the 25% percentile was about 22.4 °C, the 75% percentile was about 23.5 °C, and the median temperature was about 23 °C.
- The temperature distribution of the PP scraps was 23.3–27.2 °C, the 25% percentile was about 25.5 °C, the 75% percentile was about 26.6 °C, and the median temperature was about 26 °C.
- The temperature distribution of the EPDM scraps was 27.8–37.2 °C, the 25% percentile was about 30.2 °C, the 75% percentile was about 35.1 °C, and the median temperature was about 32 °C.
- The temperature distribution of the NBR scraps was 32.2–41.1 °C, the 25% percentile was about 34.7 °C, the 75% percentile was about 39.1 °C, and the median temperature was about 37.7 °C.
- The temperature distribution of the CR scraps was 40.2–51 °C, the 25% percentile was about 44 °C, the 75% percentile was about 48.8 °C, and the median temperature was about 45.6 °C.
- The temperature distribution of the SBR scraps was 47.6–57.6 °C, the 25% percentile was about 50.8 °C, the 75% percentile was about 55.7 °C, and the median temperature was about 52.7 °C.
- The temperature rising effects of the tested ELV polymer scraps of PVC, ABS, PP, EPDM, NBR, CR, and SBR under the irradiation power of 6 kW, respectively, are shown in Figure 13.

**Figure 14:**
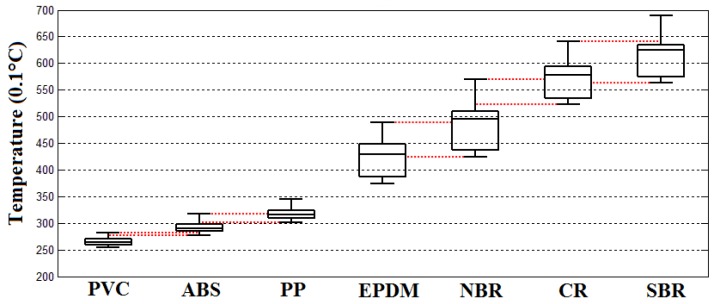
Distribution of temperature rises on tested polymer samples under microwave irradiation power of 9 kW.

The ambient temperature when this experiment was implemented was about 19.7 °C with a 6 kW microwave irradiation of 10 s. The temperature distribution features of tested scraps were:

- The temperature distribution of the PVC scraps was 23.3–26.5 °C, the 25% percentile was about 24 °C, the 75% percentile was about 25 °C, and the median temperature was about 24.4 °C.
- The temperature distribution of the ABS scraps was 25–28.3 °C, the 25% percentile was about 25.7 °C, the 75% percentile was about 27.3 °C, and the median temperature was about 26.9 °C.
- The temperature distribution of the PP scraps was 27.3–30.5 °C, the 25% percentile was about 27.8 °C, the 75% percentile was about 28.9 °C, and the median temperature was about 27.9 °C.
- The temperature distribution of the EPDM scraps was 34.8–44.9 °C, the 25% percentile was about 35.4 °C, the 75% percentile was about 41 °C, and the median temperature was about 39.2 °C.
- The temperature distribution of the NBR scraps was 37–49.6 °C, the 25% percentile was about 38.2 °C, the 75% percentile was about 45.2 °C, and the median temperature was about 42.7 °C.
- The temperature distribution of the CR scraps was 47.2–58 °C, the 25% percentile was about 49.1 °C, the 75% percentile was about 54.2 °C, and the median temperature was about 52.5 °C.
- The temperature distribution of the SBR scraps was 52.4–64 °C, the 25% percentile was about 53.3 °C, the 75% percentile was about 59.3 °C, and the median temperature was about 57.8 °C.
- The temperature rising effects of the tested ELV polymer scraps of PVC, ABS, PP, EPDM, NBR, CR, and SBR under the irradiation power of 9 kW, respectively, are shown in Figure 14.

The ambient temperature when this experiment was implemented was about 20.8 °C with a 9 kW microwave irradiation of 10 s. The temperature distribution features of tested scraps were:

- The temperature distribution of the PVC scraps was 25.5–28 °C, the 25% percentile was about 26 °C, the 75% percentile was about 27.5 °C, and the median temperature was about 27 °C.
- The temperature distribution of the ABS scraps was 27.8–32.3 °C, the 25% percentile was about 28.5 °C, the 75% percentile was about 30 °C, and the median temperature was about 29 °C.
- The temperature distribution of the PP scraps was 30.1–34.8 °C, the 25% percentile was about 31.1 °C, the 75% percentile was about 32.5 °C, and the median temperature was about 31.9 °C.
- The temperature distribution of the EPDM scraps was 37.5–49 °C, the 25% percentile was about 33.8 °C, the 75% percentile was about 45 °C, and the median temperature was about 43.1 °C.
- The temperature distribution of the NBR scraps was 42.5–57.2 °C, the 25% percentile was about 43.8 °C, the 75% percentile was about 51 °C, and the median temperature was about 49.8 °C.
- The temperature distribution of the CR scraps was 52.4–64 °C, the 25% percentile was about 53.5 °C, the 75% percentile was about 59.5 °C, and the median temperature was about 57.8 °C.
- The temperature distribution of the SBR scraps was 57–69 °C, the 25% percentile was about 57.7 °C, the 75% percentile was about 63.3 °C, and the median temperature was about 62.6 °C.

### 3.3. Analysis and Discussion

According to the results of the experiments, it was found that the microwave heating behaviors of plastics and elastomers were significantly different. Elastomers were much more sensitive to microwave radiation than plastics, which meant that the dielectric additive agents were much more active in the elastomer molecules than in the plastics. In all three power tests at 3, 6 and 9 kW, the mixture of the plastic group (PVC, PP, and ABS) and the mixture of elastomer group (EPDM, NBR, CR, and SBR) were successfully separated with an efficiency of 100%. Due to the insensitivity of plastics in microwave irradiation, their temperature variation ranges were very small. However, for the elastomers, their variation ranges were much broader than that of the plastics.

In both groups of plastics and elastomers, the temperature distribution ranges of all sorts of materials overlapped, which meant that 100% separation efficiency could not be achieved. Of course, there is no currently available technology that can totally separate these mixtures. The overlapping ranges of temperature distributions are illustrated in Figure 11, Figure 12 and Figure 13 with red dotted lines. Within the overlapping area means that at least two kinds of tested scraps that could be confused. According to the figure diagrams, there was no overlapping of three kinds of materials, which means at least the mixtures of seven kinds of polymers could be at least sorted to be mixtures of two kinds of materials with an efficiency of 100%, which could make it convenient to a large extent for the further processing and recycling of ASRs.

Of course, sorting the ASR polymer mixture only into plastic- and elastomer groups was not the aim of this research. Through further analysis of the overlap range of temperature distribution, between the overlapped ranges, there were still specific ranges where the single materials could be fully sorted to produce pure products. These ranges were named as feature temperatures for the corresponding type of polymer. The feature temperature ranges and the mass of corresponding scraps within the feature ranges are demonstrated in Table 2 as follows:

From Table 2 it can be seen that under the microwave irradiation power of 3 kW, there was a total of 170.51 kg of polymer materials that could be recognized and fully sorted, which represented about 75.04% of the mass of all the tested samples, where the sorting efficiency of PP could achieve 92.3%, SBR could achieve 84.9%, CR was 79.4%, and for ABS it was 73.3%. However, for EPDM, the sorting efficiency was 46.52%, which was lower than 50%, moreover, for NBR it was 31% and for PVC on which the heating effect was very limited, it was only 10.4%.

Under the microwave irradiation power of 6 kW, there was a total of 110.93 kg of polymer materials that could be recognized and fully sorted which represented about 48.82% mass of all the tested samples where the sorting efficiency of PP was reduced to 56.2%, for SBR it was reduced to 61.3%, for CR it was reduced to 37.3%, and for ABS it was reduced to 35%. For EPDM, the sorting efficiency was also reduced to 22%, and for NBR it was reduced to 17.6%. However, for PVC, it increased to 73.3%. With the exception of PVC, the sorting efficiency of the other six kinds of tested polymers were reduced to a large extent.

Furthermore, under the microwave irradiation power of 9 kW, a total of 83.33 kg of polymer materials could be recognized and fully sorted, which represented about 36.67% mass of all the tested samples. The sorting efficiency of PP was further reduced to 51.3%. For SBR, it was further significantly reduced to 22.6%, for CR it was reduced to 0% since the feature temperature range of NBR and SBR fully occupied the feature temperature range of CR. For ABS, it increased again to 52.1%, but was still lower than the 3 kW test. Additionally, for EPDM, the sorting efficiency increased again to 45.4%, but it was also lower than the 3 kW test. For NBR, it increased again to 27.6%, but was also lower than the 3 kW test, which was the same phenomenon as the ABS. Only for PVC did the sorting efficiency keep increasing to 86.4%.

The results of this study demonstrated that by using microwave heating effects on polymers, more than 75% of the mass of the seven kinds of ASR tested materials could be recognized and sorted. However, the sorting efficiency could not be increased by increasing the irradiation power of the microwave, on the contrary, this research indicated that by increasing the microwave power from 3 to 6 kW and then 9 kW, the sorting efficiency decreased from 75.04% to become 48.82% and further decreased to 36.67%. The highest sorting efficiency was obtained with the lowest irradiation power. The reason for this phenomenon was due to the heating behavior of the polymer materials. Through the statistics of the feature temperature distributions of tested polymers, it was found that with the increase of microwave power, the feature temperature distributions became wider. Hence, the overlapping of feature temperature ranges between adjacent materials became more serious, which limited the successful recognition during the polymer sorting process. An extreme situation was under the irradiation power of 9 kW where the recognition of CR became impossible as its feature temperature range was fully overlapped by NBR and SBR.

Although for some kinds of polymers like ABS, EPDM, and NBR, the sorting efficiency increased from 6 to 9 kW, they were still lower than the results of 3 kW. The only exception was PVC, whose sorting efficiency kept increasing with the increase of microwave power. The reason is that PVC is much more insensitive to microwave radiation than other materials, which made its temperature distribution range much less than the adjacent materials like ABS and PP, which always led its temperature distribution being separated from the others with the rise in irradiation power.

The results also indicated that the microwave sensitivities of the elastomers were much higher that the plastics, which meant that the group of tested plastic mixture and the group of tested elastomer mixture could be 100% separated. This indicates that in plastic materials, the dielectric additive agents were much more stable than those in the elastomers under microwave irradiation. Furthermore, increasing the temperature in polymers, especially in elastomers, could probably have further enhanced the activities of dielectric additives like soot, which led to a wider distribution of feature temperature and further limited the sorting efficiency. According to the theoretical model of Equations (1)–(3), and with the help of the laser-triangulation measurement, more detailed research on microwave energy attenuation and transfer behavior by transmission through each kind of tested polymers with specific thickness could be implemented.

None of the seven tested types of polymers could be fully sorted from the mixture, moreover, the results also showed that to achieve 100% sorting efficiency, the minimum variations of the feature temperature distribution between the two kinds of materials were about 5 °C and 10.5 °C for plastics and elastomers, respectively, under the irradiation power of 3 kW, as well as an irradiation period of 10 s.

## 4. Conclusions

This research verified that by using the heating effects of S-band microwave irradiation, the polymer materials in ELVs could be recognized and sorted. For a mixture consisting of seven kinds of polymers (PVC, ABS, PP, EPDM, NBR, CR, and SBR) we could achieve a maximum sorting efficiency of 75% (mass) under an irradiation power of 3 kW with irradiation period of 10 s. Furthermore, under this condition, the SBR, PP, and ABS achieved a high efficiency of recognition with 84.9%, 92.3%, and 73.3% in mass, respectively.

With this method, the plastic group (PVC, PP, and ABS) and elastomer group (EPDM, NBR, CR, and SBR) could be 100% distinguished, since the plastics were much more insensitive to microwave irradiation than the elastomers. The reason could be that the dielectric additives in elastomers have more freedom than in plastics, which led to more thermal generation through vibrations caused by the microwave. The experimental phenomenon proved that with the increase of irradiation power, the increase of feature temperature distribution ranges on elastomers became much larger than those of the plastics, which meant that in the elastomers, higher temperatures gave more freedom to the dielectric molecules.

A wider distribution of feature temperatures led to overlapping between the different polymers and further caused a significant decrease of the sorting efficiency. Therefore, in this research, we found that with the increase of irradiation power, the sorting efficiency decreased. The sorting efficiencies were 75.04%, 48.82%, and 36.67% in mass under the irradiation power of 3 kW, 6 kW, and 9 kW, respectively.

Although none of the seven kinds of tested polymers could be fully sorted from the mixture, this research has made progress and represents a major innovation in the material recycling of ELV polymers, despite the fact there still no effective method to completely sort them. The results also showed that to achieve 100% sorting efficiency, the minimum variations of feature temperature distribution between two kinds of materials are about 5 °C and 10.5 °C for plastics and elastomers, respectively, under the irradiation power of 3 kW and the irradiation period of 10 s.

Some specific polymers could also be sensitive to microwaves of other frequency domains and bands. In our future research work, the heating effects of other microwave irradiation frequencies will be studied, and the microwave acting effects on additive agents could also be studied, since the sensitivity difference of additive agents determines the heating features of the polymers and may have provide more detailed variations under different microwave frequencies.

## Figures and Tables

**Table 1:** Sampling of ASR polymers for research on domestic and foreign vehicles.

Polymer Varieties	2000–2005 Produced		2006–2010 Produced	
	Per Car Usage/kg	Sampling Parts from ELVs	Per Car Usage/kg	Sampling Parts from ELVs
SBR	48.22	Tires, shock absorption products, water pipes, brake hoses, cups	42.06	Tires, shock absorption products, metal seal ring of electrical components, water pipes, brake hoses, cups
CR	5.69	Hoses, dust cover, wiper, fan belt, synchronous belt	3.81	Hoses, dust cover, wiper, fan belt, synchronous belt
NBR	8.64	Oil filler pipe, fuel pipe, flow control hose, oil seal, film pieces	4.28	Oil filler pipe, fuel pipe, flow control hose, oil seal, film pieces
EPDM	14.29	Heater pipes, sealing strip, cables	16.46	Heater pipes, sealing strip, cables
PP	22.34	Bumpers, dashboards, lightings, panels, bonnets	31.45	Bumpers, dashboards, pedals, inner decoration
PVC	4.22	Exterior trim, cables, Upholstery, Electrical components	2.12	Cables, Electrical components
ABS	11.28	Seats, Cockpits, Interior trims, Exterior trims, lightings	12.38	Seats, lightings, bumper slides, trims

**Table 2:** Feature temperature ranges corresponding to the tested polymer masses/proportions.

Materials	3 kW		6 kW		9 kW	
	Feature Range/°C	Mass/kg (Proportion/%)	Feature Range/°C	Mass/kg (Proportion/%)	Feature Range/°C	Mass/kg (Proportion/%)
SBR	51–57.6	76.64 (84.9%)	58–64	55.33 (61.3%)	64–69	20.40 (22.6%)
CR	41.1–47.6	7.54 (79.4%)	49.6–52.4	3.54 (37.3%)	57.2–57 (Overlapped)	0 (0%)
NBR	37.2–40.2	4.0 (31.0%)	44.9−47.2	2.23 (17.6%)	49−52.4	3.57 (27.6%)
EPDM	27.8–32.2	14.21 (46.5%)	37.5−42.5	6.75 (22%)	37.5−42.5	13.96 (45.4%)
PP	24–27.2	49.65 (92.3%)	28.3−30.5	30.2 (56.2%)	32.3−34.8	27.60 (51.3%)
PVC	21–22	1.12 (10.4%)	23.3−25	4.65 (73.3%)	25.5−27.8	5.48 (86.4%)
ABS	22–23.3	17.35 (73.3%)	26.5−27.3	8.26 (35%)	28−30.1	12.32 (52.1%)
